# Screen Time and Sleep Bruxism—A Comparison Between the Present Time and the COVID-19 Pandemic

**DOI:** 10.3390/children12101396

**Published:** 2025-10-16

**Authors:** Nadezhda Mitova, Marianna Dimitrova

**Affiliations:** Department of Pediatric Dental Medicine, Faculty of Dental Medicine, Medical University, 1431 Sofia, Bulgaria; m.atanasova@fdm.mu-sofia.bg

**Keywords:** sleep bruxism, screen time, COVID-19 pandemic

## Abstract

**Highlights:**

**What are the main findings?**

- During the COVID-19 pandemic, children’s daily screen time increased, with a marked rise observed in those using screens for more than 3 h per day.
- Children with sleep bruxism spent longer periods on screens than children without bruxism (with an average difference of 32 min).

**What are the implications of the main findings?**

- Extended screen time may contribute to the development or worsening of sleep bruxism in children.
- Parents and clinicians should encourage balanced screen use and healthy sleep routines to reduce bruxism risk.

**Abstract:**

**Objectives**: The aim of this study is to assess the impact of screen time on the incidence of sleep bruxism in children during the COVID-19 pandemic. **Methods**: The parents of 266 children, aged 3–14 years, participated in the present study. They were provided with a 36-item questionnaire in order to collect data about their child’s personal information, general health, sleep bruxism, and the effects of the COVID-19 pandemic on them. The collected data were analyzed statistically using a chi-square (χ^2^) test, ANOVA with post hoc analysis (Tukey’s HSD), and a *t*-test. Results: Screen time increased significantly during the pandemic, especially among children using screens ≥180 min/day. The proportion of children spending 180–360 min/day doubled to 24.4%. Lower secondary school children had the highest screen time, with an increase of ~60 min/day during the pandemic. Smartphones were the most used device (50.8%), and on average, children with bruxism spent 32 min longer in front of screens than children without bruxism (*p* < 0.05). **Conclusions**: Daily screen use is common in children, and this increased during the COVID-19 pandemic. Children with sleep bruxism exhibit longer screen time than those without bruxism, suggesting that the former is a potential risk factor for the latter.

## 1. Introduction

### 1.1. History and Definition of Bruxism

The term “bruxism” comes from the Greek word brygmos (βρυγμóς) meaning “teeth grinding”, and it is used to describe these oral manifestations. Bruxism was first mentioned in the scientific literature by Marie and Pietkiewicz in 1907, as “la bruxomanie” [1].

According to the American Academy of Sleep Medicine (AASM), it is “a repetitive activity of the jaw muscles characterized by clenching or grinding of the teeth and/or clenching or thrusting of the lower jaw” [2,3]. The World Health Organization (WHO) also defines bruxism as a distinct disorder characterized by repetitive, rhythmic contractions of the jaw muscles that occur during sleep [4].

Bruxism can be classified according to different criteria. Teeth grinding during the day and/or while awake is defined as daytime bruxism, and grinding at night and/or while asleep is defined as sleep bruxism [3,5]. Another recently developed classification, the result of a consensus adopted by an international group of experts, uses a new diagnostic scale for both clinical and research purposes. The authors categorize bruxism using the terms possible, probable, or definite bruxism. Possible bruxism is based on data from a questionnaire completed by a patient and/or the anamnestic part of a clinical examination [2].

### 1.2. Epidemiology in Children

Sleep bruxism is more common in childhood, with a prevalence of approximately 40% in children under 11 years of age, and decreases with age. No significant difference in prevalence was found between the sexes [6]. A 2021 meta-analysis found a mean prevalence of bruxism in childhood of 31.16% [7], while Da Costa et al. reported an even higher prevalence of 47.4% [8]. The most common age group for the manifestation of bruxism is between 4 and 8 years, with a peak prevalence observed between 5 and 7 years, after which the frequency gradually decreases [9,10,11]. Pauli et al. reported a 21.46% prevalence of possible sleep bruxism, with a male predominance [12]. Similar results were presented in a Spanish study from 2023 in which 28.9% of preschoolers had sleep bruxism, with higher prevalence in 5-year-old boys [13]. According to a retrospective study, the prevalence of possible sleep bruxism in different age groups was 20.7% in children aged 0–6 years, 19.4% in children aged 7–11 years, and 14.6% in children aged 12–17 years, with no difference between genders [14].

### 1.3. Etiology and Risk Factors

The etiology of nocturnal bruxism is still not fully understood and is often considered in relation to certain associated risk factors [15]. Teeth grinding is usually associated with anatomical, physiological, or central-nervous-system-related factors [16]. Scientific evidence from experimental and clinical studies suggests that sleep bruxism is probably controlled by the central nervous system, with potential involvement of the brainstem, and its etiology is considered to be multifactorial [6,17,18]. Imbalances in certain neurotransmitters in the central nervous system (e.g., dopamine and serotonin) may also play a role in the activation of rhythmic masticatory muscle activity (RMMA) and bruxism [18]. This imbalance is associated with lifestyle factors, such as consumption of added sugar and prolonged use of electronic devices [19], as well as psychosocial factors such as anxiety, depression, and stress, which also increase the risk of teeth grinding [20]. The most common emotional factor in children who grind their teeth is stress, caused by constant nervousness and anxiety [21]. Children experiencing significant stress are more likely to develop bruxism [22].

### 1.4. Impact of the COVID-19 Pandemic and Screen Time on Bruxism

External circumstances beyond the control of parents and children can also have an impact on their daily lives. One example of this is the COVID-19 pandemic, which significantly impacted the world in 2020, leading to social isolation and long hours spent in front of electronic devices, as well as a significant change in daily routines [23,24]. According to global studies, more than 90% of children switched to distance education at some stage in the pandemic [25]. In addition, the psycho-emotional state of many children worsened during the pandemic, leading to an increase in, or reactivation of, adverse conditions, such as orofacial pain due to sleep bruxism and TMD symptoms [26,27].

On the other hand, anxiety, depression, and stress stand out as the most common problems in children caused by the pandemic [28]. Studies have shown that COVID-19 lockdowns may have been a stressor associated with bruxism in children [29,30]. Children who spend more time in front of screens have been found to have an increased frequency of teeth grinding [12,31]. A pilot study, published in 2025, showed that reducing screen time and sugar intake leads to a decrease in the frequency of bruxism when combined with a specific intervention [32]. Children who are active users of social media have been observed to spend more time on their devices, most often in the evening, before sleep, and there is evidence of increased levels of anxiety and a higher frequency of bruxism in this group [31,33,34]. The literature suggests that the changes that occurred during the COVID-19 pandemic have had a predominantly negative impact on children, both on their psycho-emotional state and on an increase in the frequency of bruxism [29,33,34,35]. According to some authors, these changes are associated with factors such as lower maternal education, freer access to electronic devices, and the presence of other sleep disorders [34]. On the other hand, in a study of 556 Brazilian children, the authors did not prove a relationship between differences in daytime screen time and the presence of sleep bruxism [19].

The accumulated data to date show that increased screen time can negatively affect children’s sleep and psycho-emotional state, which in turn could be associated with a higher risk of developing or exacerbating sleep bruxism. The available evidence is inconsistent, with some studies confirming this association, while others fail to demonstrate a clear relationship. Therefore, there is a need for a targeted study of the relationship between screen time and the manifestations of this condition.

Most studies usually link bruxism with stress, lifestyle changes, and the COVID-19 pandemic, but few studies have specifically examined the role of prolonged screen exposure as an independent, and potentially modifiable, risk factor in children. To our knowledge, no large-scale study has comprehensively evaluated the association between screen time and sleep bruxism in school-aged children during the pandemic period. By addressing this gap, the present study aims to provide new insights into behavioral risk factors and highlight opportunities for targeted prevention and early intervention with the help of the parents.

The aim of this study is to assess the impact of screen time on the incidence of sleep bruxism in children during the COVID-19 pandemic.

## 2. Materials and Methods

### 2.1. Study Population

A total of 266 children aged 3–14 years participated in this study. The participants were divided into three age groups:

- Preschool age—3–6 years;
- Primary school age—7–10 years;
- Lower secondary school age—11–14 years.

Written informed consent was obtained from all parents, in accordance with the Ethics Committee of the Medical University—Sofia (protocol #35/09.06.2023).

#### 2.1.1. Inclusion Criteria

The inclusion criteria for the study were as follows:

- Children aged 3 to 14 years whose parents properly completed the informed consent form provided;
- Children with bruxism were identified based on parental reports of teeth grinding at night, corresponding to possible bruxism [2].

It should be noted that no baseline clinical assessment or history of bruxism prior to the COVID-19 pandemic was available. Therefore, all cases were identified solely based on parental reports, which we acknowledge as a limitation of the study. Parents were also asked to indicate whether their child had shown signs of sleep bruxism prior to the COVID-19 pandemic. Only children without a consistent history of bruxism before the pandemic or those with newly reported onset during the pandemic were included in analyses evaluating pandemic-related changes.

#### 2.1.2. Exclusion Criteria

The exclusion criteria for the study were as follows:

- Children with systemic or neurological disorders that affect sleep or muscle activity (e.g., epilepsy, cerebral palsy, or parkinsonian syndromes);
- Children currently undergoing pharmacological treatment that could influence bruxism or sleep patterns;
- Children with severe craniofacial anomalies or syndromes;
- Children whose parents did not complete the questionnaire adequately or declined consent.

### 2.2. Data Collection

#### Questionnaire Method

A 36-item questionnaire was used to investigate the prevalence of sleep bruxism, risk factors for sleep bruxism, and tooth wear. The questionnaire was completed by the parents at home and included questions related to the following:

- Child and parent demographics;
- General health status and illnesses of the child;
- Dietary habits and physical activity;
- Sleep and awake bruxism;
- Sleep-related habits;
- Oral symptoms and harmful habits;
- Impact of the COVID-19 pandemic on the child’s daily life;
- Screen time.

Parents were asked to estimate their child’s total daily screen time, distinguishing between different types of activities (educational, gaming, social media, television), and, when possible, the time of use (morning/afternoon/evening). While these distinctions were encouraged, not all of the parents provided detailed breakdowns, so the primary analysis focused on total daily screen time.

The questionnaire used in the study was developed and reviewed with the active participation of dental practitioners and a psychologist, based on a review of the relevant literature. Questions were pre-tested for clarity and reliability in a small pilot group, and only clearly understood questions with reliable responses were retained.

It should be considered that the assessment of sleep bruxism relied entirely on parental reports. This approach may introduce reporting bias due to differences in parental awareness, perception, or recollections of their child’s behavior. No clinical examinations or polysomnographic recordings were conducted to confirm sleep bruxism in this cohort, which is acknowledged to be a limitation of this study, and may affect the diagnostic accuracy.

It should also be noted that the questionnaire collected information on potential confounding factors, including the child’s anxiety and stress levels, dietary habits, physical activity, pre-existing sleep disorders, and socioeconomic background. While statistical adjustment for these variables was limited, their inclusion allows for a contextual interpretation of associations between screen time and sleep bruxism. Finally, due to the cross-sectional design of the study, causal relationships cannot be definitively established.

### 2.3. Statistical Analysis

Data were analyzed using IBM^®^ SPSS^®^ Statistics 19, with a significance level set at *p* < 0.05. The following methods were applied:

- Descriptive statistics—mean ± standard deviation (SD) for continuous variables and absolute and relative frequencies for categorical variables.
- Chi-square (χ^2^) test—to evaluate associations between categorical variables, such as screen time categories and sleep bruxism status.
- One-way ANOVA with post hoc Tukey’s HSD—to compare mean screen time across age groups and identify significant pairwise differences.
- *t*-test—to compare mean daily screen time between two groups of children.

This approach enabled an assessment of trends in screen time and their relationship with sleep bruxism during the COVID-19 pandemic.

## 3. Results

**Figure 1:**
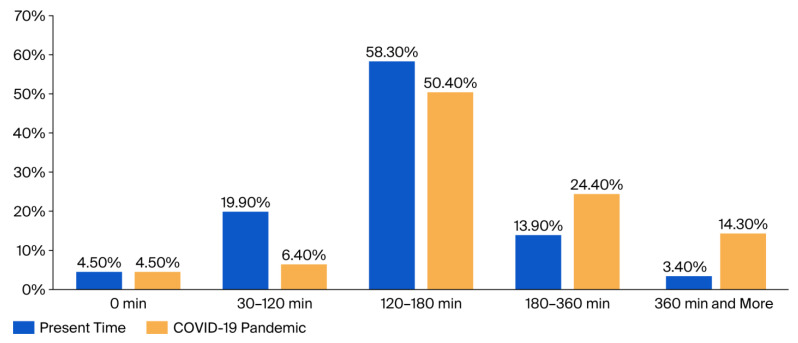
Daily screen time in children before and during the COVID-19 pandemic.

Changes in screen time during the COVID-19 pandemic were examined through the questionnaire. Figure 1 illustrates the distribution of daily screen time among the children studied.

During the pandemic, screen time increased significantly, particularly among children spending more than 180 min per day in front of screens. The proportion of children spending 360+ min per day in front of screens increased nearly fourfold, from 3.4% to 14.3%, while the proportion of children with less than 120 min/day in front of screens decreased from 24.4% to 10.9%. The proportion of children spending 180–360 min/day doubled to 24.4%.

The changes in children’s mean daily screen time during the COVID-19 pandemic and at the current time across different age groups were observed. Table 1 presents the average screen time (minutes per day) for preschool, primary-school, and lower-secondary-school children, together with the results of the ANOVA and post hoc Tukey’s HSD tests.

Preschool children currently spend an average of just over 90 min in front of a screen, and during COVID-19, the average time increased by nearly 60 min/day. In the primary school group, the increase was also about 60 min/day. The data in the table show that at the time of the study, the screen time in the lower secondary school group was the longest, and this result could also be seen during the pandemic, with a difference of about 60 min. An increase in the time spent in front of digital devices was observed in all age groups during the COVID-19 pandemic. Post hoc analysis (Tukey’s HSD) found that there is currently a statistically significant difference in screen time between the 7–10 years and 11–14 years age groups (*p* < 0.05). During the pandemic, a significant difference was also reported between the same age groups (*p* < 0.05). The differences between the 3–6-year-old group and the other age categories did not reach statistical significance due to the smaller group of participants in it (*p* > 0.05).

**Figure 2:**
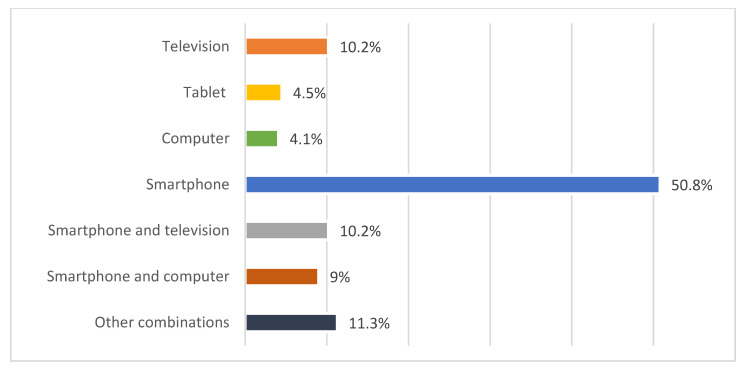
Distribution of digital devices most frequently used by children.

Parental reports on device usage revealed that smartphones were the most frequently used device among children (50.8%), likely due to their easy accessibility. More than 30% of children use more than one device per day, potentially increasing their total daily screen time (Figure 2).

Next, the differences in the daily screen time between children with and without sleep bruxism during the pandemic period were evaluated. The received data indicated that children with bruxism were more likely to spend longer periods in front of screens compared to children without bruxism. The effect of the COVID-19 pandemic on daily screen time (minutes) in children with and without sleep bruxism is summarized in Table 2.

For the analysis of the acquired data, the χ^2^ test was used to compare categorical distributions of screen time. A higher proportion of children with bruxism were in the longer screen time categories (180+ min/day: 27.8%) compared to children without bruxism (14.6%), and excessive screen time (360+ min/day) was more common among children with bruxism (9.3%) than those without (1.9%). An independent samples *t*-test was used to compare the mean daily screen time between groups. The mean daily screen time was 167.2 ± 102.2 min in children with bruxism versus 135.3 ± 79.3 min in children without bruxism (χ^2^ = 14.67, *p* = 0.005; t = 2.484, *p* = 0.014). These results indicate that children with sleep bruxism spent significantly more time in front of screens, suggesting a positive association between prolonged screen exposure and the presence of bruxism.

## 4. Discussion

Due to the variety of risk factors associated with bruxism and the significant changes to daily life during the COVID-19 pandemic, the focus of the present study is to investigate the impact of digital device use during the pandemic on sleep bruxism. Studies show that screen-time affects dopamine neurotransmission [36], which may also be involved in the etiology of sleep bruxism [18]. Together, the serotonergic and dopaminergic systems regulate the homeostasis of the inhibitory–stimulatory balance of neurophysiological processes like respiration, muscle tone, and stress [18,36]. The dopaminergic system is responsible for skeletal muscle activity, cognitive activity, depression, neuroendocrine control, and neurological mechanisms of reward [36]. These neurobiological connections motivated our interest in studying the potential impact of screen time on sleep bruxism. Distance learning during the COVID-19 pandemic led to a significant increase in the time spent by children in front of screens [24,25].

In the present study, participants were divided into five groups according to their daily duration of screen time, from 0 to over 360 min. The data show that the most common duration is between 120 and 180 min per day, both at the time of the study (58.3%) and during the pandemic (50.4%). However, it was found that screen time significantly increased during the pandemic period, especially in children who spent over 180 min per day in front of a device. The proportion of children using digital devices for more than 360 min per day increased from 3.4% before the pandemic to 14.3% during it. The average duration of screen time increased from 141.8 min to 205 min per day. Preschoolers, primary school children, and lower secondary school children spent an average of 150 min, 187 min, and 226.8 min a day, respectively, using digital devices. This represents an average increase of about 60 min across all age groups. The most frequently used device is the smartphone (50.8%), with over 30% of children using more than one device [19].

A significant difference was found when comparing children with and without bruxism—27.8% of children with bruxism fell into the group with over 180 min of screen time, compared to 14.6% of those without bruxism. The mean daily screen time in children with bruxism was 167.2 min, 32 min longer than in children without bruxism (135.3 min), which was statistically significant.

The results of the current study are consistent with the data from other authors who reported a significant increase in screen time among children during the pandemic [24,33]. In addition, some studies indicate a possible relationship between extended time in front of digital devices and an increased incidence of bruxism [31]. For example, in Gurunathan’s study, 100% of the children studied who spent more than 2 h in front of a device showed signs of bruxism, and all children who spent less than 1 h did not have any [37]. The observed trends may be associated with a higher incidence of bruxism, possibly due to reduced sleep quality, increased stress, or less physical activity.

The prolonged use of digital devices can cause alterations in circadian rhythm and influence cognitive development and functioning, mood regulation, social performance, attention, and behavior, thus becoming a factor possibly associated with sleep disturbances in children [38,39]. Physiological and psychological mechanisms, including the effect of screen-induced dopamine changes and stress due to prolonged screen time, could explain the association between screen exposure and sleep bruxism [38].

In this study, it was observed that children with bruxism exhibit a prolonged screen usage, suggesting that clinicians should include specific recommendations for families, such as removing electronic media from children’s bedrooms, including televisions, computers, tablets, and smartphones, to reduce potential negative effects on children’s health [40].

Another mechanism that can be associated with the development of sleep bruxism due to prolonged screen time usage is the low levels of physical activity that inevitably accompany it. Physical activity can reduce stress and prevent parafunctional habits by decreasing muscle tension, hypertension, asthma, heart arrhythmia, and related complications [40,41,42]. There is a mild association between the presence of sleep bruxism and the practice of physical activity in children of ages between 4 and 8 years of age. Children who played more had reduced incidence of bruxism, and children who spent more time using their gadgets were more prone to bruxism than children who spent less time on them [37].

### 4.1. Limitations

This study has several limitations that should be considered while interpreting the results. First, it has a cross-sectional design, which is not very appropriate for establishing causal inferences between screen time and sleep bruxism. Nevertheless, the findings provide valuable insights and can guide future longitudinal studies to better understand the relationship between screen time and sleep bruxism.

It is important to note that, due to the cross-sectional design, causal relationships between screen time and sleep bruxism cannot be definitively established. However, there are plausible biological mechanisms that may explain the observed association. Prolonged screen use may increase stress levels, alter dopamine and serotonin neurotransmission, and disrupt circadian rhythms, which are all factors that potentially contribute to the onset or exacerbation of sleep bruxism in children [38]. The use of digital devices in the evening may interfere with sleep quality and amplify parafunctional behaviors [38]. From a clinical perspective, these findings suggest that pediatric dentists and parents should monitor and limit children’s screen time, particularly before bedtime, and encourage balanced daily routines, including physical activity, adequate sleep, and stress-reducing activities. Preventive strategies could include removing electronic devices from bedrooms, setting clear usage limits, and promoting offline leisure activities. Future longitudinal studies are warranted to confirm these associations, differentiate between types of screen activities, and examine their timing and intensity in relation to sleep bruxism [31,32]. Objective measurements, such as clinical examinations or electromyographic recordings, could strengthen the evidence for causal relationships and help guide more targeted interventions [3].

Second, the assessment of both sleep bruxism and screen time relied on parental reports. While this approach is feasible in large pediatric samples, it is subject to recall bias and parental perception, which may influence the true prevalence. In particular, the diagnosis of bruxism was based on parental observations alone rather than clinical or instrumental confirmation, which limits the diagnostic accuracy [2,19,31].

This emphasizes that no baseline clinical assessment or history of bruxism prior to the COVID-19 pandemic was available. Therefore, all cases were identified solely based on parental reports, which is acknowledged as a limitation. Parents were also asked to indicate whether their child had shown signs of sleep bruxism prior to the COVID-19 pandemic. Only children without a consistent history of bruxism before the pandemic or those with newly reported onset during the pandemic were included in analyses evaluating pandemic-related changes [2,19].

Third, parents reported daily screen time without considering the period of the day in which the children used the screen (evening or daytime) or the activity performed (educational, entertainment, social media, or a combination). There is a wide diversity of activities that can be performed on each electronic device, and especially during the COVID-19 pandemic, they were probably mainly used for distance learning. These factors may influence sleep quality and bruxism differently, but were not evaluated in this study [19,38,39].

Parents were asked to estimate the total daily screen time, distinguishing between different types of activities (educational, gaming, social media, television), and when possible, the times of use (morning/afternoon/evening). While making these distinctions was encouraged, not all parents provided detailed breakdowns, so the primary analysis focused on the total daily screen time [19].

### 4.2. Future Research Directions

Future research should aim to overcome these limitations by using longitudinal designs that allow for an assessment of the causal relationship between screen time and the onset or exacerbation of sleep bruxism. It is also important for future research to differentiate between types of screen activities (passive viewing, interactive gaming, educational use, social media) and to assess the times of use, particularly in the evening hours before sleep. These variables may indicate which digital behaviors are most strongly associated with bruxism. For more precise results, future studies should include objective diagnostic methods, such as clinical dental examinations and electromyographic recordings, to confirm the diagnosis of sleep bruxism [3].

This is the first Bulgarian study that evaluates the influence of screen time on sleep bruxism in children and the impact of the COVID-19 pandemic on both these factors. The findings can provide recommendations for establishing healthy habits in children, emphasizing the role of clinicians in advising parents on appropriate screen use. Parents should be aware of the connection between prolonged screen time and teeth grinding [19,38,39].

## 5. Conclusions

The present study demonstrates that daily screen time is highly prevalent among children and tends to increase during exceptional circumstances, such as the COVID-19 pandemic. Moreover, children diagnosed with sleep bruxism were found to have significantly longer screen time compared to children without bruxism. These results emphasize a notable difference in screen usage patterns between the two groups, highlighting the need for increased awareness regarding screen time habits in pediatric populations, particularly among those with sleep-related disorders.

## Figures and Tables

**Table 1:** Mean daily screen time (minutes) in children by age group at the current time and during the COVID-19 pandemic.

Screen Time	At the Current Time	During the Pandemic
Age Group	Mean ± SD	Mean ± SD
3–6 years	93.3 ± 67.8	150 ± 99.5
7–10 years	126.6 ± 72.3	187 ± 110.8
11–14 years	160.2 ± 93.9	226.8 ± 151.2
Total	141.8 ± 85.2	205 ± 133.1
ANOVA	F = 6.811 *p* = 0.001	F = 3.734 *p* = 0.025
Tukey’s HSD	7–10 _Γ_. vs. 11–14 _Γ_. (*p* = 0.004)	7–10 _Γ_. vs. 11–14 _Γ_. (*p* = 0.045)

**Table 2:** Daily screen time (minutes) during the COVID-19 pandemic in children with and without sleep bruxism.

Children Screen Time (Minutes per Day)	Without Bruxism		With Bruxism	
	*N*	%	*N*	%
0 min	11	5.2%	1	1.9%
30–120 min	38	17.9%	13	24.1%
120–180 min	130	61.3%	28	51.9%
180–360 min	27	12.7%	10	18.5%
360 min and more	4	1.9%	5	9.3%
Total	212	100%	54	100%
χ^2^ = 14.67, *p* = 0.005				
Screen time (mean value)	*N*	Mean ± SD	*N*	Mean ± SD
	212	135.3 ± 79.3	54	167.2 ± 102.2
t = 2.484 *p* = 0.014				

## Data Availability

The data presented in this study are available on request from the corresponding author due to privacy and ethical restrictions.

## Abbreviations

The following abbreviations are used in this manuscript:

AASM	American Academy of Sleep Medicine
WHO	World Health Organization
RMMA	Rhythmic masticatory muscle activity
COVID-19	Coronavirus disease 2019
TMD	Temporomandibular Disorders
ANOVA	Analysis of Variance
Tukey’s HSD	Tukey’s Honest Significant Difference
